# Macroporous latex biomembrane from *Hancornia* speciosa modulates the inflammatory process and has a debridement effect on wound healing in rats

**DOI:** 10.1590/acb385323

**Published:** 2023-10-23

**Authors:** Kassya Lopes Epaminondas Martins, Marcelo Martins Thomaz, Lais Nogueira Magno, Marina Clare Vinaud, Luciane Madureira Almeida, Pablo José Gonçalves, Ruy de Souza Lino

**Affiliations:** 1 Universidade Federal de Goiás – Tropical Pathology and Public Health Institute – Goiânia (GO) – Brazil.; 2 Instituto Master de Ensino Presidente Antônio Carlos – Medicine School – Itumbiara (GO) – Brazil.; 3 Universidade Federal de Goiás – Physics Institute – Goiânia (GO) – Brazil.; 4 Universidade Federal de Goiás – Anápolis (GO) – Brazil.

**Keywords:** Biocompatible Materials, Wound Healing, Anti-Inflammatory Agents, Debridement

## Abstract

**Purpose:**

The angiogenic, osteogenic and anti-inflammatory activity of latex of *Hancornia speciosa* has been evidenced and indicates pharmacological potential with great applicability in the health area, especially in the wound healing process. The present work aimed to compare the effects of the *H. speciosa* macroporous latex biomembrane with saline on wound healing.

**Methods:**

Forty-three Wistar rats were submitted to excisional wound induction procedure and divided into groups according to treatment: saline (G1), and macroporous biomembrane (G2). The animals were euthanized at three, seven, 14, and 21 days after injury induction (DAI), and three animals were used for the debridement test. Morphometric, macroscopic, and microscopic analyses of general pathological processes were performed.

**Results:**

The macroporous biomembrane minimized necrosis and inflammation during the inflammatory and proliferative phases of the healing process, confirmed by the lower intensity of the crust and the debridement effect. In addition, the wounds treated with the macroporous biomembrane presented greater contraction rates in all the experimental periods analyzed.

**Conclusions:**

The macroporous biomembrane presents angiogenic, anti-inflammatory and debridement effects, contributing to the healing process, and can be considered a potentially promising new biomaterial to be used as a dressing.

## Introduction

Several strategies have been proposed for the treatment of wounds, aiming to assist in tissue repair, among them polymers, plant extracts, physical dressings, laser, hyperbaric oxygen, dermal matrices, negative pressure therapy, doxycycline, minocycline, and alginate^1^-^5^. However, although there are currently several types of dressings commercially available, there is still no product considered a gold standard for the treatment of wounds, especially for complex chronic wounds^4^, often lacking effective treatment^6^-^8^.

A product, in order to be considered effective in the treatment of wounds, must be easily removed, provide comfort to the patient, do not require frequent changes, be cost-effective, improve epidermal migration, induce blood vessels formation and synthesis of connective tissue, provide or keep the wound surface with ideal humidity and the periphery of the lesion dry and protected, have easy application and adapt to different parts of the body^4^,^6^-^8^. Also, the humid wound environment is important to induce the ideal context for physiological healing, in addition to limiting the inflammatory reaction, necrosis, lesion progression, and scar size^9^,^10^.

However, there are no drugs that meet all the necessary criteria for the ideal treatment of chronic injuries^11^,^12^. Therefore, popular medicinal culture has been used as source of plant derived medicines^13^. In this context, latex has stood out. Estimates show that there are 12 to 35 thousand lactiferous species, and only a few have been evaluated regarding their pharmacological importance^14^,^15^. Among them, the most explored and known is the rubber tree, from which the latex extracted presents angiogenic and healing properties. Recently, some reports have shown that some lactiferous plants, especially *Hancornia speciosa*, a Brazilian native plant, has important properties for wound healing^15^-^18^.

The latex of *H. speciosa* has been used against trauma, inflammation, diarrhea, tuberculosis, ulcers, herpes, and its leaf tea against menstrual cramps^18^. In addition, it has been used to treat inflammatory diseases, hypertension, dermatitis, liver disease, diabetes and gastric disorders^19^. *H. speciosa* leaves have compounds that aid in the control of blood pressure alterations^20^ and diabetes^21^. Also, in *in-vitro* wound healing essays the ethanolic extract of the leaves promoted an increase in cell migration and proliferation of fibroblasts^22^, while in *in-vivo* essays it has shown an anti-inflammatory action^23^ and to be not cytotoxic nor genotoxic^24^,^25^.

The biomembranes obtained from the latex of *H. speciosa* have angiogenic^25^ and osteogenic potential^26^,^27^, assisting in wound healing. These biomembranes can incorporate antibacterial drugs into their matrix, releasing them in a controlled manner, thus allowing the combination of angiogenic, anti-inflammatory, and antibacterial properties^28^. Also, the incorporation of silver nanoparticles in latex caused the inhibition of the *Staphylococcus aureus* biofilm formation without impairing the process of inflammation and tissue repair^16^.

Recently, a macroporous biomembrane was developed from *H. speciosa* latex for use in wound healing and patented as “latex-based macroporous biomembrane for use as a dressing in wound healing processes” number BR 10 2019 003829 2^29^. The present study aimed to describe and compare the debridement and healing effect on excisional wounds in rats submitted to treatment with macroporous biomembrane.

## Methods

### Preparation of the macroporous biomembrane

The biomembrane was produced from latex collected at the experimental station of the Universidade Estadual de Goiás, in the municipality of Ipameri, Goiás, Brazil. Latex was collected from a cut^30^ in the bark and stored in a sterile container. The cut was about 10 cm long and 0.5 cm deep. The collected latex was diluted in purified water in a 1:1 ratio (water:latex) to prevent clotting. Then, it was centrifuged at 3,000 g for 5 minutes at 4°C. Ten mL of the supernatant was deposited in a petri dish (10.00 ± 0.05 cm in diameter) and incubated at 45°C until complete polymerization (72 hours). Subsequently, the obtained biomembrane was cut to a size of 2 × 2 cm, perforated using a metallic cannula to form pores of 2 mm in diameter as described in the patent “latex-based macroporous biomembrane for use as a dressing in wound healing processes” number BR 10 2019 003829 2^29^. Finally, it was packed in surgical grade paper and sterilized at the Energy and Nuclear Research Institute (IPEN), in São Paulo, São Paulo, Brazil, by gamma radiation through a Gammacell 220 cobalt (Co60) source, at a dose of 25 kGy^25^. Then, it is available to wound healing assays.

### Ethical considerations

This study was conducted according to ethical standards after approval by the Ethics Committee on the Use of Animals of the Pontifícia Universidade Católica de Goiás (protocol 16-01) and followed the regulations determined in the guide produced by the Nacional Council of Control of Animal Experimentation^31^,^32^. Three animals were kept per cage in order to maintain their well-being, with occlusive dressing capable of protecting the injuries. The cages were made of polypropylene, lined with wood shavings, and cleaned twice a week. The animals were previously acclimated. The luminosity, the temperature, the noise intensity, and the relative humidity of the air were those of the general environment. Water and commercial ration for the species were offered *ad libitum* to the animals.

### Experimental groups

For the accomplishment of this study, 43 male rats of the *Rattus norvegicus albinus* species, with 2-3 months of age and body weight between 200 and 300 grams at the beginning of the experiment, were used. Among them, 40 animals were randomly assigned to the groups according to the treatment:

- G1: control group, which received NaCl 0.9% treatment, with 20 animals;
- G2: test group, which was treated with macroporous biomembrane, with 20 animals.

For analysis of the proposed parameters, five animals from each experimental group were euthanized at three, seven, 14, and 21 days after injury induction (DAI) for morphometric and anatomopathological analysis of the injured area. An additional test was also performed with three animals to allow the analysis of the debridement effect of the macroporous biomembrane.

The number of animals was determined through previous studies that used this same experimental model, in addition to books and articles that determine the calculation of the number of animals^30^. Equation 1 was used to calculate the number of animals:


$$n=1+\left[2C^{*}(s/d)2\right]$$ (1)

Where: n = number of animals; C = 10.51 and significance level (α = 0.05; the chance of considering two different groups when they do not are); s = 0.2; Acceptable standard deviation: 20%; d = 0.5; Expected difference between groups: 50%; n = 1 + [2*10,51*(0,2/0,5)2]; n = 4.36 animals per group.

### Excisional lesion induction, cleaning, and treatment

Initially, the animals were weighed and anesthetized intraperitoneally with a solution of 10% ketamine and 2% xylazine at the dosage of 1 mL/kg (injectable Dopalen Sespo Indústria e Comércio, Brasil; and Xilazin, Syntec do Brasil). Eye lubrication with saline was also performed to prevent corneal ulceration. After being anesthetized, the animals were placed in prone position. Trichotomy, and antisepsis of the area to be injured were performed with gauze soaked in 70% ethanol. An acrylic mold measuring 2 × 2 cm in area and a pen were used to define the area to be injured. The lesion was induced through a superficial cut with a scalpel, followed by the deepening of the cut to the limit of the skin, without damaging the muscle tissue. Subsequently, the tissue fragment (± 1.5 mm) was gently removed with the aid of scalpel and scissors, preserving the panniculus carnosus. The lesion area was cleaned with sterile gauze soaked in saline (NaCl 0.9%).

**Figure 1:**
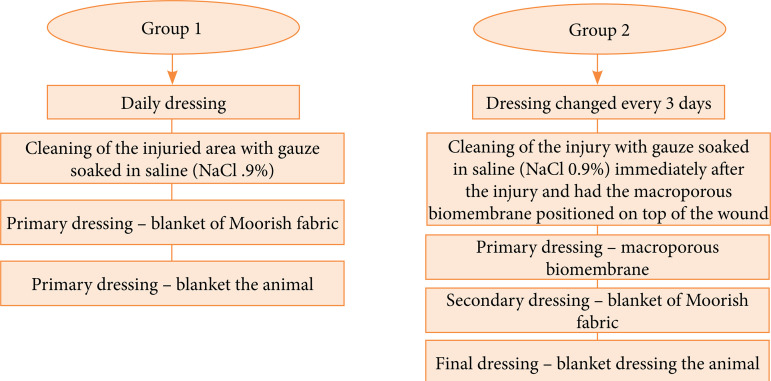
Schematic representation of the procedures performed in group 1, control, and group 2, treated with the macroporous biomembrane.

Source: Elaborated by the authors.

The procedure was performed as follows:

- G1 received a cleaning of the injured area with gauze soaked in saline (NaCl 0.9%). This procedure was repeated daily up to 21 days after the injury induction (Fig. 1).
- G2 received a cleaning of the injury with gauze soaked in saline (NaCl 0.9%) immediately after the injury induction and had the macroporous biomembrane positioned on top of the wound in order to cover the entire injury area with the membrane, without crossing the injury borders. The membrane was changed at every three days up to 21 days after the injury induction (Fig. 1). The periodicity of the biomembrane change was determine according to the observed saturation of the dressing, which is considered a good characteristic for a dressing due to less manipulation of the injury, causing less pain and stress for the animal.

Throughout the experiment, the injuries were protected by an occlusive dressing in order to keep the wound moist, closed and prevent additional injuries caused by the other animals in the cage. The composition of this dressing was a blanket of Moorish fabric, so that it dressed the entire back of the animal, with lashing on the sides, which was changed according to the group. Immediately after the injury, and on the proposed days, the wounds were cleaned with sterile gauze soaked in saline (NaCl 0.9%) and treated according to the group.

### Procedure for euthanasia

On experimental days 3, 7, 14, and 21, the animals were euthanized with an intraperitoneal injection of 2.5% thiopental (Thiopentax, Cristália Produtos Químicos Farmacêuticos, Brazil) at the dosage of 120 mg/kg^33^ Afterwards, the animals were placed on a board for the photographic record, using a Sony Cyber-shot DSC-H300 camera (Sony. Followed by the removal of the skin and subcutaneous tissue fragment from the lesion area, using a scalpel, scissors, and forceps, preserving the muscular plane, the fragments were fixed in 10% buffered formaldehyde.

### Macroscopic analysis

The wound healing phases were evaluated on the proposed experimental days as described ahead: at three DAI the inflammatory phase, at seven DAI the proliferative phase and at 14 and 21 DAI the maturation phase was evaluated. The photos of the injuries allowed the analysis of the presence of crust in a semi-quantitative way, according to the following criteria: absent, discreet (involvement of up to 25% of the area), moderate (from 26 to 50% of the area), and accentuated (over 50% of the area).

The photographs were taken using a tripod at 20 cm from the animal, after the euthanasia. Also, the macroscopic images were accompanied with a scale that allowed the calibration of all measurement’s parameters. The photographs were transferred to a computer and analyzed using the Image J 1.3.1 software. To determine the degree of contraction of the wound area, the formula proposed by Oliveira et al.^34^ was used (Eq. 2):


Relative wound contraction(%)=[(initial injured area−contracted injured area)/initial injured area]×100 (2)

### Microscopic analysis

The fragments of each wound, fixed in 10% buffered formaldehyde, were processed, and stained with hematoxylin and eosin (HE), according to Luna^35^, for histological evaluation. The following general pathological processes in the dermis were analyzed: necrosis / crust, which was recognized through morphology and observation of cellular debris, fibrin, inflammatory polymorphonuclear infiltrate, and mononuclear infiltrate, which were identified through cellular morphology, blood vessels quantification, fibroblasts, and matrix deposition. These aspects were classified in a semi-quantitative manner, according to the following criteria: absent (score = 0); discreet (score = 1) with impairment of up to 25% of the area; moderate (score = 2), from 26 to 50% of area impairment; and accentuated (score = 3) above 50% of area impairment^36^.

### Statistical analysis

Statistical analysis was performed using the Sigma Stat 2.3 software. All variables were tested for normal distribution and homogeneous variance. When the data distribution was normal, parametric tests were used, such as T test. When the data distribution was not normal, non-parametric tests were used, such as Mann-Whitney’s. The observed differences were considered significant when p < 0.05.

## Results

### Wound contraction and macroscopic analysis

**Figure 2:**
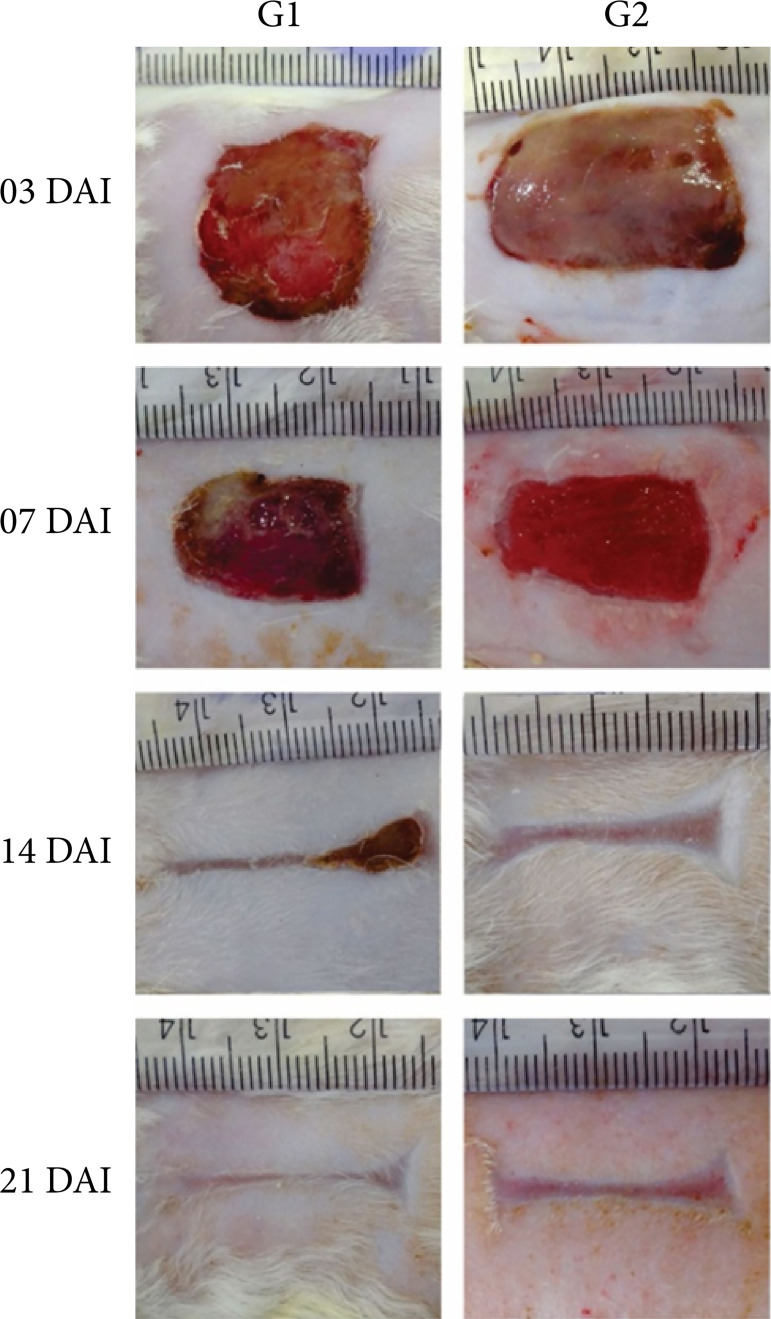
Macroscopic evolution of the excisional wounds experimentally induced in rats at three, seven, 14, and 21 DAI. Scale in mm.

**Figure 3:**
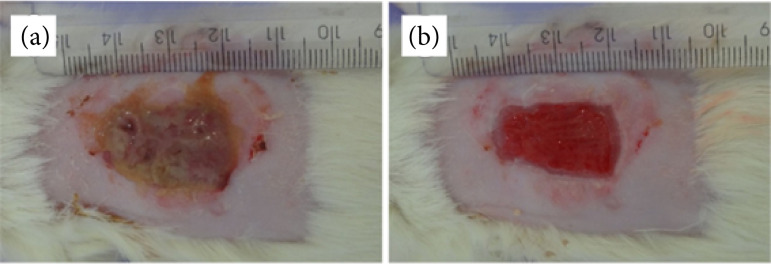
Macroscopic aspect of the excisional wound at seven days after the injury in group 2, treated with the macroporous biomembrane. **(a)** Before the cleaning with saline solution (NaCl 0.9%). **(b)** After the cleaning showing the total removal of the viscous substance and evidencing the debridement effect of the biomembrane. Scale in mm.

Source: Elaborated by the authors.

**Figure 4:**
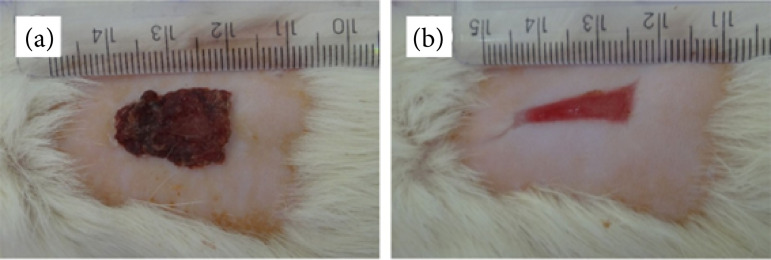
Excisional wound experimentally induced in rats. **(a)** Wound treated with saline solution (NaCl 0.9%) for eight days after the injury induction showing the crust formation through all the wound extension. **(b)** Wound treated with the macroporous biomembrane for three days without crust. Scale in mm.

Source: Elaborated by the authors.

The wound contraction was significantly higher in G2 than in G1 at three, seven, and 21 DAI (p < 0.05). At 21 DAI, it was possible to observe that 80% of the wounds were completely closed in both studied groups (Table 1, Figs. 2–4).

**Table 1:** Wound contraction (percentage) of excisional wounds experimentally induced in rats and treated with macroporous biomembrane of *Hancornia speciosa*.

Days after injury induction	G1	G2	P-value
	% (Mean ± SD)	% (Mean ± SD)	
3	22.3 ± 9.5	36.9 ± 6.6	0.030*
7	42.4 ± 13.7	62.8 ± 4.3	0.013*
14	90.2 ± 2.6	92.5 ± 1.1	0.102
21	90.3 ± 1.8	94.8 ± 2.0	0.006*

SD: standard deviation; G1: control group treated with saline (NaCl 0.9%); G2: group treated with macroporous biomembrane of *Hancornia speciosa*. Statistical test: Student’s t test;

* statistically significant difference. Source: Elaborated by the authors.

G1: control group treated with saline solution (NaCl 0.9%); G2: group treated with the macroporous biomembrane; DAI: days after the injury induction. Source: Elaborated by the authors.

In the inflammatory and proliferative phase, at three and seven DAI, respectively, less intensity of crust was observed in G2 when compared to G1 (p < 0.05) (Table 2).

At seven DAI, all the wounds in G2 were shown to be covered with a viscous substance (Fig. 3a). The biomembrane showed ability to absorb the exudate, preventing adherence to the dressing, and was easily removed with cleaning (Fig. 3b), allowing the visualization of the granulation tissue formed below. No crust formation was observed, demonstrating the autolytic debridement effect of this product without damaging the newly formed tissue.

**Table 2:** Crust formation in excisional wounds experimentally induced in rats and treated with macroporous biomembrane of *Hancornia speciosa*.

DAI	G1 M (min–max)	G2 M (min–max)	p-value
3	2.0 (2.0–3.0)	0.0 (0.0–0.0)	0.029*
7	2.0 (1.0–3.0)	0.0 (0.0–0.0)	0.016*
14	1.0 (0.0–2.0)	0.0 (0.0–0.0)	0.190
21	0.0 (0.0–0.0)	0.0 (0.0–0.0)	1.000

DAI: days after the injury induction; M: median; (min–max): minimum–maximum; G1: control group treated with saline (NaCl 0.9%); G2: group treated with macroporous biomembrane of *Hancornia speciosa*;

* statistically significant difference. The statistical analysis considered a semi-quantitative analysis as follows: 0 – absent; 1 – discrete; 2 – moderate; 3 – accentuated. Statistical test used: Mann-Whitney. Source: Elaborated by the authors.

### Debridement test

In order to confirm the debridement effect observed in G2, an additional test was carried out to verify the use of this material on a consistent and firmly adhered crust. For this, the animals (n = 3) were submitted to the standard injury procedure, and the wounds were cleaned with saline (NaCl 0.9%) until a crust was formed. At eight DAI (Fig. 4a), the macroporous biomembrane was applied for three days. It was possible to observe that the entire crust had been debrided, the lesion was smaller, without necrosis and with evident granulation tissue (Fig. 4b).

### Microscopic analysis

**Figure 5:**
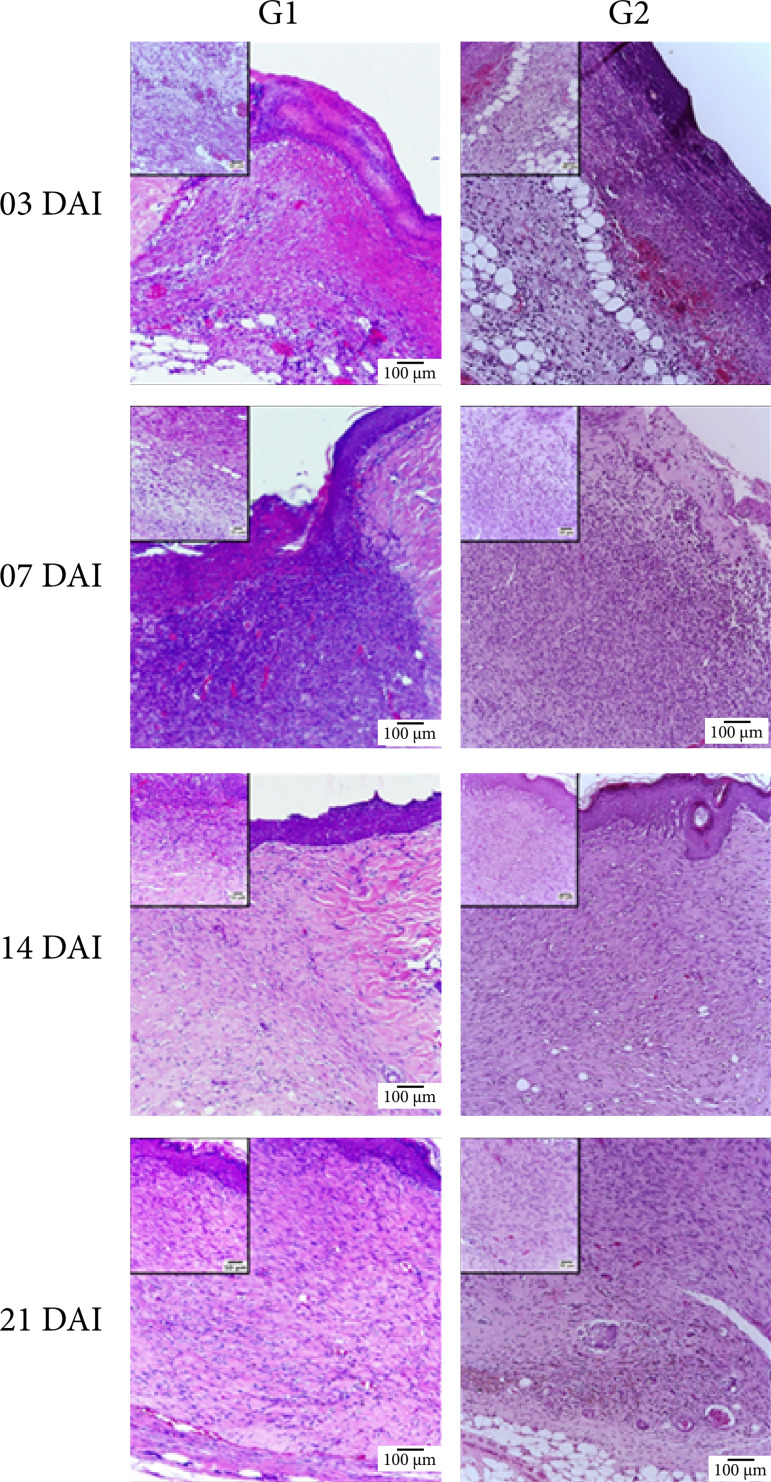
Photomicrograph of the skin of rats which suffered the experimental induction of an excisional wound at three, seven, 14, and 21 DAI. Scale: 100 μm. Insert: 50 μm.

DAI: days after the injury induction; G1: control group, which received treatment with saline solution (NaCl0.9%); G2: group treated with the macroporous biomembrane. Stain: hematoxiline and eosin. Source: Elaborated by the authors.

In the inflammatory phase, at three DAI, G2 presented less intensity (p < 0.05) of necrosis and polymorphonuclear cells infiltrate than G1 (Table 3 and Fig. 5).

**Table 3:** General pathologic processes observed in excisional wounds experimentally induced in rats and treated with macroporous biomembrane of *Hancornia speciosa*.

General pathologic processes	DAI	G1 Median (min–max)	G2 Median (min–max)	p-value
Necrosis/crust	3	3.0 (3.0–3.0)	2.0 (1.0–3.0)	0.032*
	7	2.0 (1.0–3.0)	0.0 (0.0–1.0)	0.008*
	14	0.0 (0.0–0.0)	0.0 (0.0–1.0)	0.690
	21	0.0 (0.0–0.0)	0.0 (0.0–0.0)	1.000
Polymorphonuclear cells infiltrate	3	3.0 (3.0–3.0)	2.0 (1.0–2.0)	0.008*
	7	2.0 (1.0–2.0)	0.0 (0.0–1.0)	0.016*
	14	0.0 (0.0–0.0)	0.0 (0.0–1.0)	0.690
	21	0.0 (0.0–0.0)	0.0 (0.0–1.0)	1.000
Mononuclear cells infiltrate	3	2.0 (2.0–2.0)	2.0 (1.0–2.0)	1.000
	7	3.0 (3.0–3.0)	3.0 (3.0–3.0)	1.000
	14	2.0 (1.0–3.0)	1.0 (1.0–2.0)	0.010*
	21	1.0 (1.0–2.0)	1.0 (1.0–2.0)	0.010*
Blood vessels quantification	3	1.0 (0.0–1.0)	1.0 (0.0–1.0)	0.690
	7	2.0 (1.0–2.0)	3.0 (2.0–3.0)	0.016*
	14	3.0 (3.0–3.0)	2.0 (2.0–2.0)	0.008*
	21	1.0 (0.0–2.0)	1.0 (1.0–2.0)	0.841
Fibroblast	3	1.0 (1.0–1.0)	1.0 (1.0–1.0)	1.000
	7	3.0 (3.0–3.0)	2.0 (2.0–2.0)	0.008*
	14	3.0 (3.0–3.0)	3.0 (3.0–3.0)	1.000
	21	3.0 (3.0–3.0)	2.0 (2.0–3.0)	0.032*

DAI: days after the injury induction; M: median; (min–max): minimum–maximum; G1: control group treated with saline (NaCl 0.9%); G2: group treated with macroporous biomembrane of *Hancornia speciosa*;

* statistically significant difference. The statistical analysis considered a semi-quantitative analysis as follows: 0 – absent; 1 – discrete; 2 – moderate; 3 – accentuated. Statistical test used: Mann-Whitney. Source: Elaborated by the authors.

In the proliferative phase, at seven DAI, there was less intensity (p < 0.05) of necrosis, polymorphonuclear infiltrate and fibroblasts in G2 when compared to G1. Also, G2 biomembrane group presented greater intensity of blood vessels quantification (p < 0.05) when compared to G1 (Table 3 and Fig. 5).

In the maturation phase, at 14 DAI, there was less intensity of blood vessels quantification in G2 when compared to G1. At 21 DAI, there was less intensity (p < 0.05) of fibroblast in G2 when compared to G1 (Table 3 and Fig. 5).

There was no significant difference in the fibrin and collagen analysis between the experimental groups throughout the experimental days (data not shown).

## Discussion

This study addressed the evaluation of the healing of excisional wounds experimentally induced in Wistar rats, using as treatment the *H. speciosa* macroporous latex biomembrane compared to a control group, in which only cleaning with saline (NaCl 0.9%) was performed.

Regards to physicochemical *H. speciosa* biomembrane characterization used in the present study, different analyses were previously published. In relation to its composition, *H. speciosa* latex does not differ significantly from other latex and is aqueous polydispersed system containing around 50% of water, 40–45% weight of rubber molecules (cis-1,4-polyisoprene is the main component) and 4–5% weight of nonrubber constituents (protein, fatty acids, carbohydrates, and sugar)^37^. Concerning nonrubber constituents, the low-protein content of *H. speciosa* latex may be responsible for its non-allergenic potential^27^,^37^.

In addition, phytochemical characterization identified chlorogenic acids, naringenin-7-O-glucoside, catechin and procyanidin in *H. speciosa* latex composition^17^,^26^. Chlorogenic acids present antioxidant, anti-mutagenic, and anti-inflammatory activities and can modulate metabolic pathways associated with wound healing^17^,^26^.

Regards to the physical properties, the *H. speciosa* biomembrane have elastomeric behavior with good elasticity, low mechanical hysteresis and high elongation capacity^27^. Flexibility is a desirable characteristic for development of wound healing dressings as the dressing can be adjusted to the injury bed. In addition, *H. speciosa* biomembrane contains micrometric pores in its surface^2^, which allow nutrients permeation, cells adherence, bones adhesiveness and others^27^. It was observed the porosity and the size of micropores can be controlled by membranes preparation method^38^. Since the permeation rate increases with pore size^39^, macropores on the order of millimeters could facilitate the leakage of fluid from the wound. This allows the wound to remain in a moist environment but without excess exudate.

Then, in addition to micropores naturally present in biomembrane surface, the biomembranes used in this study were mechanically perforated to obtain macropores of 2 mm in diameter. All those *H. speciosa* biomembrane properties have showed that latex is a promising biomaterial in wound healing as it stimulates blood vessels formation and presents biocompatibility to organic tissues^16^,^17^,^25^,^27^. The results of this study were demonstrated through the analysis of general macroscopic and microscopic pathological processes.

In this study, the wounds were surgically induced on the animals’ backs and during the entire follow-up period occluded with a dressing made of sterile Moorish fabric, allowing the absorption of exudate, aeration of the tissue, maintenance of humidity and, above all, protection avoiding additional injuries by the animals of the same cage. The dressings also prevented contamination and kept the biomembrane fixed in the appropriate place. According to the literature, the use of an occlusive dressing has advantages, such as: preventing the wound from drying out and forming a crust, which can hinder the formation of the new epithelium^7^, increase the absorption of the applied substances, soothe pain, stimulating growth factors, enzymes necessary for debridement and protecting the wound^9^. Moreover, occlusion should allow gas exchange to occur between the wound bed and the environment 4.

Currently, the importance of keeping the wound environment hydrated by maintaining the ideal moisture has been recognized so that dressings must maintain it without causing maceration of the wound bed^39^. In this study, an occlusive dressing was used, which prevented dryness, but the humid environment was completely assured using the macroporous biomembrane. A biomaterial was developed for use in wound healing, presented in the form of a flexible biomembrane, which can be adapted to different parts of the body, of varying sizes and porous, allowing the release of exudate.

During the inflammatory phase, it was possible to observe in this study that the treatment with macroporous biomembrane associated with cleaning with saline inhibited the formation of crust. Possibly, this can be attributed to the maintenance of ideal humidity, without causing maceration^40^. On the other hand, the cleaning with saline dried the wound, causing it to form a crust, which exposed it to bacterial contamination and infection^41^.

At seven days, in the proliferative phase, the macroscopic analysis in this study showed the debridement effect of the macroporous biomembrane through the regression of the crust intensity and by the granulation tissue under development. This result was confirmed by the histopathological study in which, on that same experimental day, there was less intensity of necrosis / crust and marked increase in blood vessels quantification, a fundamental process for the formation of new tissue^42^. Debridement was performed without causing additional injuries, and it can be of great applicability in humans, since in chronic necrotic lesions there is a need for debridement to allow the normal development of the healing process^43^.

Still in the proliferative phase observed in our results, the greater intensity of blood vessels quantification in the group that used the macroporous biomembrane corroborated a study of the latex of *H. speciosa* in the chorioallantoic membrane model, in which it induced marked increase in blood vessels quantification similar to Regederm (a product based on latex from *Hevea brasiliensis*) and greater than the negative and inhibitory control^25^. According to the literature, the blood vessels formation is essential for tissue growth, cellular reproduction, and wound repair, allowing proliferation, migration, regulation and differentiation of vessel cells^44^, migration of macrophages and fibroblasts to the wound site, enabling the production of extracellular matrix and later the collagen that will form the scar^45^.

The angiogenic activity of *H. speciosa* has been studied in a chorioallantoic membrane model that shows that this effect is observed because of the serum fraction^17^, which presents secondary metabolites, enzymes and luteoid compounds^25^. It has been also shown that this serum fraction stimulates the expression of proangiogenic factors such as vascular endothelial growth factor and MMP2 and stimulates the remodeling of the extracellular matrix^17^.

The results obtained after the appliance of the macroporous biomembrane, in the present study, were similar to the ones described previously^46^, using the latex biomembrane of *H. brasiliensis* in surgical cutaneous wounds in Wistar rats. These authors also observed the prevention of crust formation during all phases of the healing process and the visual aspect of the lesion with the formation of a viscous layer protecting and maintaining the moisture of the wound. This debridement potential has been also verified in other studies using *H. brasiliensis* latex biomembrane in decubitus eschar^47^,^48^ and patients with chronic venous ulcers^49^.

Therefore, the presentation of the dressing in the form of biomembrane showed an important factor to enable debridement since the use of latex of *H. brasiliensis* in the form of gel-cream did not have a significant influence on the healing of acute lesions experimentally induced in rats^50^,^51^.

The use of *H. speciosa* latex in biomembrane form in order to maintain the humid environment in the wound bed and to prevent necrosis / crust and inflammation is supported by the lower intensity of inflammation observed in this study, as less polymorphonuclear infiltrate in the fragments removed from animals treated with the macroporous biomembrane and by the debridement potential observed in G2. Therefore, for the environment to be considered ideal for the development of the healing process, the wound must be kept moist, especially in excision wounds^2^.

The lesser intensity of migration of polymorphonuclear leukocytes during the initial stages of the healing process observed in this study can be attributed to the probable anti-inflammatory action of the macroporous biomembrane. These findings are in accordance with Marinho et al.^23^, which showed that the latex of *H. speciosa* inhibited nitric oxide, PGE2 and cytokines interleukin-6 and tumor necrosis factor, resulting in an anti-inflammatory action by influencing both migration and activation of inflammatory cells. This anti-inflammatory effect was maintained, in the present study, until the final stages of the experimental analysis with decreased migration of mononuclear leukocytes during the maturation phase, showing fine regulation throughout the healing process.

In our study, the wounds treated with the macroporous biomembrane had a higher percentage of contraction at three, seven, and 21 days, demonstrating a regulation of the remodeling process of the extracellular matrix. This process can be attributed to the interaction between chlorogenic acids and MMP2, which are present in greater concentration in the serum fraction of the latex from *H. speciosa* 17.

The *H. speciosa* latex biomembrane can also be used in combination with other products, as it has already demonstrated that the incorporation of silver nanoparticles leads to high anti-biofilm efficiency without changing the blood vessels formation induction capacity^16^, showing a promising herbal medicine in the field of complex wound healing.

## Conclusion

In conclusion, the macroporous biomembrane proved to be an important biomaterial for wound healing, leading to greater intensity of wound contraction. In the inflammatory and proliferative phase, it led to less formation of necrosis / crust, showed debridement and possible anti-inflammatory action, while in the maturation phase it induced decreased induction of blood vessels formation, inflammatory infiltrate, and migration of fibroblasts.

## Data Availability

The data will be available upon request.
